# Unravelling the impact of dental workforce training and education programmes on policy evolution: a mixed-method study protocol

**DOI:** 10.1186/s12961-023-01048-9

**Published:** 2023-09-12

**Authors:** Galvin Sim Siang Lin, Shu Meng Goh, Mohd Haikal Muhamad Halil

**Affiliations:** 1 Department of Dental Materials, Faculty of Dentistry, Asian Institute of Medicine, Science and Technology (AIMST) University, 08100 Bedong, Kedah Malaysia; 2 Department of Public Health Medicine, Faculty of Medicine and Health Science, Universiti Sabah Malaysia, 88400 Kota Kinabalu, Sabah Malaysia; 3 Department of Restorative Dentistry, Kulliyyah of Dentistry, International Islamic University Malaysia, 25200 Kuantan, Pahang Malaysia

**Keywords:** Dental services, Education programme, Healthcare reform, Health policy, Stakeholder participation

## Abstract

**Background:**

The dental workforce plays a crucial role in delivering quality oral healthcare services, requiring continuous training and education to meet evolving professional demands. Understanding the impact of dental workforce training and education programmes on policy evolution is essential for refining existing policies, implementing evidence-based reforms and ensuring the growth of the dental profession. Therefore, this study protocol aims to assess the influence of dental workforce training and education programmes on policy evolution in Malaysia.

**Methods:**

A mixed-method research design will be employed, combining quantitative surveys and qualitative interviews. Stakeholder theory and policy change models will form the theoretical framework of the study. Participants from various stakeholder groups will be recruited using purposive sampling. Data collection will involve surveys and one-on-one semi-structured interviews. Descriptive statistics, inferential analysis and thematic analysis will be used to analyse the data. Integration of quantitative and qualitative data will be used to provide a comprehensive understanding of the data.

**Discussion:**

This study will shed light on factors influencing policy decisions related to dental education and workforce development in Malaysia. The findings will inform evidence-based decision-making, guide the enhancement of dental education programmes and improve the quality of oral healthcare services. Challenges related to participant recruitment and data collection should be considered, and the study’s unique contribution to the existing body of knowledge in the Malaysian context will be discussed.

## Background

The dental workforce plays a pivotal role in delivering quality oral healthcare services to individuals and communities [1]. As the field of dentistry continues to evolve, dental professionals must remain well-equipped with the latest knowledge, skills and best practices to meet the ever-changing demands of the profession [2]. Dental workforce training and education programmes serve as vital conduits for equipping dental practitioners with the necessary competencies and fostering their continued professional development [3, 4]. Amidst the dynamic landscape of dentistry, it is essential to understand the impact of dental workforce training and education programmes on policy evolution. These programmes not only influence the skills and capabilities of individual practitioners but also contribute to shaping broader policy frameworks that govern dental practice, workforce regulation and patient care standards [5, 6]. In the context of dental workforce training and education programmes, policy evolution refers to the changes and reforms made in the policies that govern the dental profession, dental education and workforce regulation [7]. These policies may cover areas such as curriculum development, licensing and certification requirements, accreditation standards, scope of practice, continuing education requirements and quality assurance mechanisms. Hence, gaining comprehensive insights into the relationship between dental education programmes and policy development is crucial for refining existing policies, implementing evidence-based reforms and ensuring the continued growth and adaptability of the dental profession.

Malaysia is a nation with a middle-class income and an increasingly ageing population [8]. Both public and private institutions and agencies are responsible for the oral healthcare system of the country. The dental workforce training and education programmes in Malaysia have undergone significant developments in recent years [9, 10], reflecting the country’s commitment in improving oral healthcare services and addressing the evolving needs of the population. It also contributes to the promotion of oral health and the prevention and treatment of dental diseases in the nation [11]. Dental workforce training and education programmes in Malaysia are primarily conducted in dental schools and institutions of higher learning [12]. These programmes encompass undergraduate education, postgraduate education (coursework based, research based or mixed mode) and continuing professional development initiatives. Undergraduate dental education programs typically span 5 years and provide dental students with a comprehensive foundation in basic sciences, clinical skills and professional ethics [13]. On the other hand, coursework-based postgraduate education programmes offer specialization opportunities in various dental disciplines, allowing dentists to acquire advanced knowledge and skills in specialized areas of practice [14]. The research-based postgraduate programmes allow dental professionals to develop in-depth knowledge and skills in conducting cutting-edge dental research. Furthermore, continuing professional development programmes focus on lifelong learning and provide opportunities for dental professionals to enhance their skills and stay updated with the latest advancements in the field [15].

Understanding the impact of these programmes on policy evolution is crucial for policy-makers, dental educators, dental practitioners, allied dental professionals (dental therapists, dental nurses and dental technicians) and regulatory bodies to ensure the availability of a skilled and competent dental workforce to deliver quality oral healthcare services across the nation. To the best of the author’s knowledge, this study represents the first of its kind to be conducted in Malaysia. Although a previous study has explored key stakeholders’ views on the drivers for change in the Malaysian dental workforce [8], little research has focused on examining the impact of dental workforce training and education programmes on the current national policy. Therefore, this study protocol aims to fill this gap by assessing the influence of dental workforce training and education programmes on policy evolution across different regions, dental institutions and regulatory bodies in Malaysia. Furthermore, it also aimed to identify the challenges faced by these programmes and propose future directions for their enhancement. The research questions are as follows: (1) What are the perceptions of the stakeholders in the current dental workforce training and educational programmes in Malaysia? (2) How do dental workforce training and education programmes in Malaysia impact policy evolution within the dental profession? (3) What challenges do these programmes face in terms of shaping and refining national dental policies?

## Methods

### Ethics

The study protocol has received ethical approval from the institutional research ethics committee, indicated by the ethical approval code AUHEC/FOD/06/20/04/2023. This approval ensures that the study adheres to ethical guidelines and safeguards the rights and confidentiality of the participants. Informed consent will be obtained from all key informants, ensuring that they have a clear understanding of the study’s purpose, procedures and potential risks or benefits. To maintain utmost confidentiality, data collection will be anonymous, meaning that participant identities will not be linked to their responses.

### Study design

This study will employ a mixed-method research design, combining both quantitative surveys and qualitative interviews. By utilizing both methods, the study can gather comprehensive data on stakeholder perceptions regarding the impact of dental workforce training and education programmes on policy evolution. This mixed-method approach allows for a more in-depth and holistic understanding of the topic, capturing both numerical data and rich qualitative insights.

### Theoretical framework

The present study will be guided by the theoretical framework of stakeholder theory [16] and policy change models [17, 18]. By using the stakeholder theory, the study will emphasize the perspectives, interests and interactions of various stakeholders involved in dental education and workforce development. These stakeholders include dental educators, dental students, policy-makers, regulatory bodies and dental professionals. By considering their viewpoints, the study can examine how different stakeholders influence policy decisions and reforms. Additionally, policy change models, such as the advocacy coalition framework [17] and the policy process model [18], will be used to understand the drivers and processes of policy evolution in the context of dental education and workforce development. These models can help to identify key actors, policy networks and factors that shape policy decisions, including how dental education programmes contribute to policy changes.

### Sample size calculation

The sample size determination was executed utilizing G*Power software, version 3.1.9.6 (Universitat Kiel, Germany; February 2020), employing the *F*-test, with α value of 0.05 and β value of 0.80. Given the paucity of data in prior literature regarding the precise population count of dental healthcare professionals in Malaysia, employing parameter estimates (standard deviation of differences, mean differences between pairs) from previous research to guide our sample size calculation was infeasible. However, in response, we adopted Cohen’s guideline to ascertain the effect size for this study [19]. Assuming a substantial Cohen’s *d* (*d* = 0.4), we employed this as our effect size. We categorized the main stakeholders into four main groups: dental educators (comprising both public and private institutions), practising dentists (comprising general dental practitioners and dental specialists), dental students (encompassing undergraduate and postgraduate students) and allied dental professionals (including dental nurses and dental technicians). A minimum sample size of 68 subjects per group is necessitated. Accounting for a projected attrition (drop-out) rate of 15%, the total required sample size amounts to 313 individuals.

### Participant recruitment

Participants for this study will be recruited from key stakeholder groups, including dental educators from both public and private institutions, undergraduate and postgraduate dental students, general dental practitioners, dental specialists and allied dental professionals who are currently working in either public or private sectors and hold a Malaysian citizenship. A purposive sampling strategy (maximum variation) will be employed to ensure diverse representation within each stakeholder group across the nation. This sampling approach allows for a deliberate selection of participants who possess relevant knowledge and experiences related to dental workforce training and education programmes, ensuring that the study captures a wide range of perspectives. Participants will be invited to participate via email or telephone calls. If there is no response after 1 week, a follow-up email will be sent. All participants will be given consent to participate in the study and they must hold Malaysian nationality.

### Data collection

- Quantitative surveys
  The reporting for the present study will follow the consensus-based checklist for reporting of survey studies (CROSS) [20]. To gather quantitative data, surveys will be developed based on an extensive literature review and input from experts in the dental workforce training and policy. The items in the survey will be content validated by the experts and undergo pilot testing before the survey can be distributed to the participants. These surveys will assess participants’ perceptions of the impact of dental workforce training and education programs on policy evolution. Likert-scale items, which involve respondents indicating their level of agreement or disagreement on a five-point scale, will be included in the surveys. Additionally, open-ended questions will be incorporated to capture qualitative data and allow participants to provide detailed explanations or insights.
- Qualitative interviews
  The reporting for the present study will adhere to the consolidated criteria for reporting qualitative research (COREQ) recommendations [21]. One-on-one semi-structured interviews will be conducted with a subset of participants to obtain in-depth insights into their perceptions and experiences. These interviews will explore stakeholders’ perspectives on policy evolution, the influence of training and education programmes, and suggestions for policy enhancements. The interviewer will be an expert who had prior experience in conducting qualitative research. The time and place of the interview will be arranged considering the participants’ choices. The interviews will be audio-recorded and later transcribed by two investigators independently to facilitate qualitative analysis. The interviews will continue until data saturation when no new information or codes can be found. Member-check will also be performed. Interview transcripts will be distributed to participants who agreed to provide feedback for their comments.

### Data analysis

- Quantitative analysis
  The survey data will undergo descriptive statistics and inferential analysis to identify patterns, trends and statistical associations. This analysis will provide a quantitative overview of stakeholders’ perceptions regarding the impact of dental workforce training and education programmes on policy evolution. Descriptive statistics summarize the data, while inferential analysis examines relationships or differences between variables. Cronbach’s alpha and confirmatory factor analysis will be performed to assess the reliability and validity of questionnaire items.
- Qualitative analysis
  Thematic analysis will be employed to analyse the qualitative interview data. The transcripts of the interviews will be systematically analysed through coding, identifying recurring themes, patterns and meaningful categories. The investigators will then discuss and finalize the themes and subthemes. Discrepancies will be resolved by consensus among all investigators. This analysis aims to gain a deep understanding of stakeholders’ perspectives and provide rich qualitative insights into the impact of dental workforce training and education programmes on policy evolution.

### Integration of quantitative and qualitative data

The study will employ a convergent design approach to integrate the quantitative and qualitative data. Triangulation will be performed by comparing and contrasting the findings from both data sets. By integrating the findings, the study aims to develop a comprehensive understanding of the impact of dental workforce training and education programmes on policy evolution, combining numerical trends with contextualized insights from stakeholders.

## Discussion

This study aims to evaluate the influence of dental workforce training and education programs on policy evolution in Malaysia. The knowledge generated from this study can foster dialogue and collaboration between dental stakeholders, enabling collective efforts to advance the dental workforce and enhance the overall quality of oral healthcare delivery. This research intends to contribute to the optimization of dental workforce training and education programmes, ultimately leading to improved patient outcomes and more effective policies that shape the future of dental practice. This discussion section will focus on the reasons why this study protocol needs to be conducted, the potential benefits it can bring, the challenges that may arise and a comparison with related studies conducted in other countries. First, by exploring the perspectives of various stakeholders, including dental educators, students, practitioners and policy-makers, this study can shed light on the diverse factors that shape policy decisions and reforms related to dental education and workforce development.

Second, the findings will provide valuable insights into the effectiveness of dental workforce training and education programmes in Malaysia and their impact on policy evolution. The study’s results can highlight areas of strength and areas needing improvement in dental education programmes in Malaysia. If stakeholders perceive that certain aspects of dental education are not effectively preparing graduates for their roles or that there are gaps in the curriculum, policy-makers and educational institutions can use this information to reform and enhance the dental education system. For instance, if the study finds that there is a need for more focus on certain specialized areas of practice, dental schools can consider revising their curricula to address these needs. This could lead to better-trained dental professionals who are equipped to meet the evolving demands of oral healthcare [22].

Moreover, the insights gained from the study could contribute to enhancing the overall quality of oral healthcare services in Malaysia. If the study reveals areas where dental workforce training and education programmes are positively influencing policy evolution, these practices could be expanded or replicated to ensure that dental professionals are well prepared to provide high-quality care. By aligning dental education with policy objectives, oral healthcare services can become more patient centred, evidence based and efficient [23]. While the study is specific to Malaysia, the insights gained from it could potentially be adapted to other countries with similar healthcare systems and dental education structures. If the study identifies successful strategies or challenges that resonate with broader issues faced by dental education systems, policy-makers and educational institutions in other countries might find the findings relevant to their own contexts.

In addition, employing a mixed-method research design, it captures both quantitative and qualitative data, allowing for a comprehensive understanding of the topic [24]. The use of surveys with Likert-scale items provides quantitative data, enabling the identification of patterns and trends in stakeholders’ perceptions of the impact of dental workforce training and education programmes on policy evolution. The incorporation of open-ended questions in the surveys and qualitative interviews further enriches the data by providing detailed explanations and insights from participants. Furthermore, this study protocol utilizes a theoretical framework consisting of stakeholder theory and policy change models to explore the perspectives, interests and interactions of various stakeholders involved in dental education and workforce development. By incorporating their viewpoints, this study can provide a holistic understanding of the influence of different stakeholders on policy decisions and reforms. The use of both advocacy coalition framework and the policy process model, allows for an examination of the drivers and processes of policy evolution in the context of dental education and workforce development. These frameworks will enhance the theoretical underpinning of the study and contribute to a deeper understanding of the factors shaping policy decisions.

However, conducting this study may encounter certain challenges. Firstly, participant recruitment from diverse stakeholder groups across the nation may be challenging due to logistical constraints and limited resources [8]. The recruitment of participants through purposive sampling might introduce selection bias, as participants may not fully represent the entire spectrum of stakeholder views and experiences. Therefore, ensuring adequate representation within each stakeholder group is crucial to capture a wide range of perspectives. Secondly, due to the nature of voluntary participation, those who agree to participate might hold stronger opinions or experiences related to the study topic, potentially leading to overrepresentation of certain perspectives. Furthermore, social desirability bias could influence participants’ responses in surveys and interviews, as they might provide answers they perceive as socially acceptable or expected, rather than their true views. Another potential challenge is the need to balance the depth and breadth of data collection. The study aims to gather comprehensive data through surveys and interviews, but the time and resources required for data collection, analysis and interpretation should be carefully managed [25, 26].

Undeniably, disparities between private and public sectors in dental workforce training and education programmes are crucial to acknowledge [27]. These discrepancies can manifest in grant distribution, learning opportunities and social demographic factors. Public institutions often have greater financial resources, potentially leading to unequal access to grants and funding, resulting in varied programme development and infrastructure. This disparity may enable public institutions to provide more advanced learning experiences and specialized training. Additionally, resource and infrastructure differences can lead to disparities in learning opportunities, affecting the skill sets acquired by dental students. Moreover, social demographic factors come into play as public institutions attract different student demographics due to factors such as tuition fees and reputation. This could create diversity gaps and affect representation in the dental workforce. Both public and private sector interests can influence policy evolution, possibly leading to differing policy advocacy and decisions [8]. Addressing these discrepancies is vital to ensure equitable access to quality dental education. Recognizing these differences is crucial for a balanced and effective dental workforce that can contribute to oral healthcare needs.

In comparing this study protocol with related studies conducted in other countries, it is important to acknowledge that each context has its unique characteristics and healthcare system. While several studies have explored the impact of dental training and education programmes [28–30], the specific policy frameworks, educational approaches and stakeholder dynamics may differ across countries. Therefore, this study offers the opportunity to contribute to the existing body of knowledge by providing insights specific to the Malaysian context. By examining the experiences and perspectives of stakeholders in Malaysia, this study can offer localized insights that can inform policy decisions and reforms in the country’s dental education system.

## Data Availability

All data generated or analysed during this study will be included in the published article.

## Abbreviations

COREQ: Consolidated criteria for reporting qualitative research
CROSS: Consensus-based checklist for reporting of survey studies
